# Cognitive Persistence and Executive Function in the Multilingual Brain During Aging

**DOI:** 10.3389/fpsyg.2020.568702

**Published:** 2020-09-03

**Authors:** Susan Teubner-Rhodes

**Affiliations:** Department of Psychological Sciences, Auburn University, Auburn, AL, United States

**Keywords:** aging, bilingual, multilingual, cognitive control, persistence < motivation/engagement, cingulo-opercular network, cognitive reserve

## Abstract

Researchers have debated the extent to which the experience of speaking more than two languages induces long-term neuroplasticity that protects multilinguals from the adverse cognitive effects of aging. In this review, I propose a novel theory that multilingualism affects cognitive persistence, the application of effort to improve performance on challenging tasks. I review recent evidence demonstrating that the cingulo-opercular network, consisting of the bilateral inferior frontal gyrus (IFG) and dorsal anterior cingulate cortex (dACC), supports cognitive persistence. I then show that this same network is involved in multilingual language control and changes with multilingual language experience. While both early and late multilinguals exhibit differences in the cingulo-opercular network compared to monolinguals, I find that early multilinguals have a pattern of decreased dACC activity and increased left IFG activity that may enable more efficient cognitive control, whereas late multilinguals show larger dACC responses to conflict that may be associated with higher cognitive persistence. I further demonstrate that multilingual effects on the cingulo-opercular network are present in older adults and have been implicated in the mitigation of cognitive symptoms in age-related neurodegenerative disorders. Finally, I argue that mixed results in the literature are due, in part, to the confound between cognitive persistence and ability in most executive function tasks, and I provide guidance for separating these processes in future research.

## Introduction

Numerous studies have been devoted to the question of the extent to which the experience of speaking two or more languages improves cognitive control, the ability to suppress irrelevant information in the service of goal-oriented performance (for review, see Bialystok, 2017). While the evidence for an effect of multilingualism on cognitive control among younger adults is mixed (Hilchey and Klein, 2011; Paap et al., 2014; Lehtonen et al., 2018), some research suggests that multilingual effects on cognition may be more extensive and reliable among older adult populations (Bialystok et al., 2004; Hilchey and Klein, 2011; Gold et al., 2013b). As cognitive control naturally declines with age, a lifetime of experience managing multiple languages may protect the brain from decline or allow multilinguals to achieve better levels of performance with similar levels of neural degeneration (Bialystok et al., 2009).

Based on the extant literature on the effect of multilingualism on cognitive control across the lifespan, I propose a novel theory that multilingualism enhances cognitive persistence, the ability to apply effort to overcome difficulty (Teubner-Rhodes et al., 2017), which is enacted by increased reactivity of the cingulo-opercular system to performance decrements and/or conflict. Critically, I argue that multilingualism differentially affects cognitive control and cognitive persistence depending on age of language acquisition and language proficiency, providing the source of variability in findings regarding the neuroprotective effects of multilingualism. Higher cognitive persistence would enable multilinguals to perform at higher levels than monolinguals as the brain declines with age by supporting the increased effort necessary to overcome decrements in cognitive function. This would have the effect of making age-related declines in performance more gradual because individuals with higher persistence can apply effort to offset increased task difficulty. In contrast, higher cognitive control would enable multilinguals to perform at higher levels than monolinguals but would not affect the rate of decline with age.

I first review evidence that multilingual language control taps and changes the cingulo-opercular network that supports cognitive persistence. This review helps explain how differential influences of multilingualism on cognitive persistence and cognitive control may have led to mixed findings in the literature. I then discuss findings that multilingualism affects the cingulo-opercular network during cognitive aging, which suggest that cognitive persistence is a possible protective mechanism against age-related declines in performance. Finally, I provide guidance for researchers wishing to distinguish between cognitive persistence and cognitive control and outline specific hypotheses about how differential multilingual experiences affect each function during cognitive aging.

While much of the literature has been devoted to the question of the effect of multilingualism on cognitive control, cognitive persistence has received less attention. Measuring cognitive persistence in multilingual cognitive aging research offers several benefits that will advance the field: 1) There are several available assessments of cognitive persistence that researchers can add to their existing protocols to co-vary out the contribution of effort to performance on cognitive control tasks. This will allow researchers to separate the effects of multilingualism on cognitive persistence versus cognitive control to reconcile mixed findings in the literature. 2) Cognitive persistence can be measured before cognitive declines occur to test the hypothesis that individuals with higher persistence will exhibit smaller age-related performance decrements. 3) Cognitive persistence provides a specific neurocognitive mechanism that could protect multilinguals from adverse aging effects. Namely, higher cognitive persistence improves performance by increasing reactivity of the cingulo-opercular network and exertion of effort in response to task difficulty, which increases as individuals age.

## Cognitive Persistence in Multilingualism

### What Is Cognitive Persistence?

**FIGURE 1:**
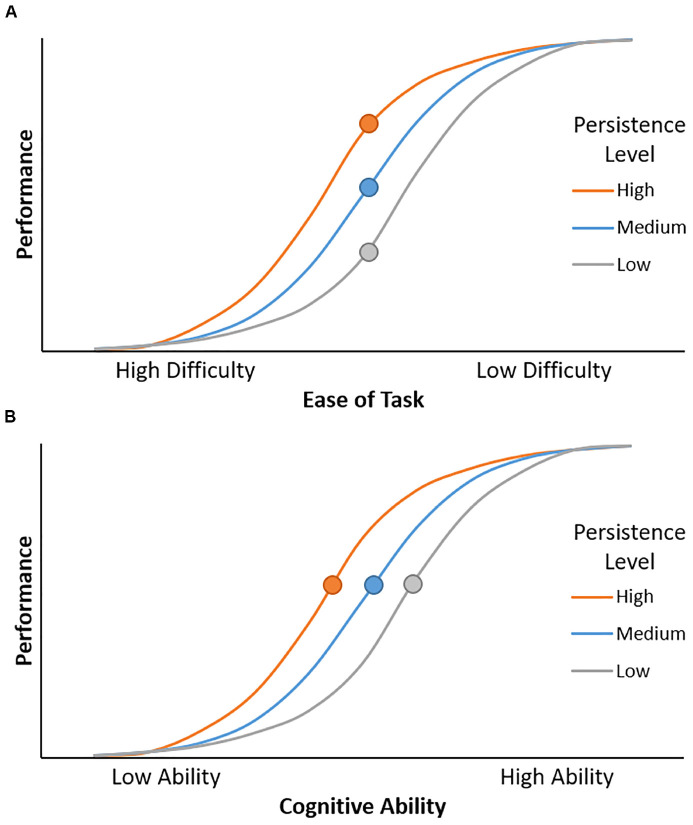
**(A)** Cognitive persistence modulates the relationship between ease of task and performance. Performance increases as the task becomes easier. Given equivalent task difficulty, individuals with higher levels of persistence have better performance. This effect is maximal at intermediate levels of task difficulty (circles). **(B)** Cognitive persistence modulates the relationship between cognitive ability and performance. Performance increases as cognitive ability increases, but high cognitive persistence can offset low cognitive ability. Specifically, an individual with relatively low cognitive ability but high persistence (orange circle) can achieve the same level of performance as individuals with higher ability but lower persistence (blue and gray circles).

As cognitive persistence reflects individual differences in the willingness and ability to sustain effort, it is particularly important for performing well during challenging tasks. For instance, imagine trying to follow a conversation in a noisy environment. An individual with low persistence may give up and stop listening due to the difficulty of trying to recognize the speech in background noise – they are unwilling or unable to invest the effort necessary to understand the speech. In contrast, an individual with higher persistence may increase their effort investment in order to successfully understand the conversation. Importantly, cognitive persistence should only affect performance when a task is difficult, yet possible. If the task exceeds an individual’s ability level, then their performance will be low regardless of how hard they work at the task. Similarly, persistence does not affect performance when a task is too easy, because performance is high even without applying effort. Thus, cognitive persistence will have the greatest impact on performance for effortful tasks with an intermediate difficulty level (see Figure 1A).

Task difficulty is determined by two factors, task demand and cognitive ability. Task demand is fixed across individuals, determined by experimental manipulations such as stimulus conflict and memory load. In contrast, cognitive ability varies across individuals. The same task will be easier and thus yield higher performance for individuals with higher cognitive ability. Put simply, individuals who have higher cognitive control will have better performance on cognitive control tasks. However, for decades, psychologists have pointed out performance is not just a function of cognitive ability, but also the individual’s drive to succeed and the effort they devote to a task (Wechsler, 1950; Militello et al., 2006). An individual who is less capable to perform a task but has high cognitive persistence may invest substantial effort into a task, enabling better performance than typically observed for their ability level. Thus, it is possible for cognitive persistence to offset some deficits in cognitive ability (Figure 1B), including age-related cognitive declines.

How exactly would cognitive persistence compensate for age-related declines in cognitive control ability? Individuals with higher cognitive persistence and their lower persistence counterparts may perform comparably on cognitive control tasks during young adulthood, when their cognitive control ability is relatively high and they are able to perform these tasks without exerting much effort. However, as cognitive control ability declines with age, these tasks become more difficult and require more effort to perform well. Individuals with lower persistence are expected to show large age-related declines in performance, as they exert less effort in response to increasing task difficulty (Figure 1A). In contrast, higher persistence individuals invest more effort as the task becomes more difficult with age, resulting in smaller / delayed performance declines. In this way, having higher cognitive persistence could counteract performance decrements that result from age-related declines in cognitive control, enabling individuals to perform nearly as well as younger adults despite having poorer cognitive function.

### Cognitive Persistence and Language Control

How is cognitive persistence relevant to multilingualism? Several models of multilingual language control propose that multilinguals use inhibitory control to suppress their competing language(s) when speaking in their other language (for review, see Abutalebi and Green, 2008; Kroll et al., 2008; Green and Abutalebi, 2013; Declerck and Philipp, 2015). Neuroimaging has shown that multilinguals engage overlapping neural resources for conflict control and language switching (Abutalebi et al., 2012), suggesting that multilinguals use domain-general inhibitory control resources to suppress one language and activate the other. Lower proficiency multilinguals in particular may need inhibitory control to suppress their dominant L1 when speaking in a weaker L2 or L3 (Green, 2011; Declerck and Philipp, 2015). This idea was initially supported by asymmetrical switch costs, the finding that bilinguals were slower to switch from their L2 to their L1 than vice versa during a cued picture naming task (Meuter and Allport, 1999). This paradoxical result suggested that bilinguals exhibited stronger suppression of their L1 when speaking in L2, so that bilinguals need to reactivate L1 when switching back to it. Further research demonstrated that language switch costs increased when the target language had been used recently. Specifically, trilinguals exhibit a larger cost when switching from L2 to L1 if they had used L1 on the previous trial than if they had used L3 (i.e., L1-L2-L1 vs. L3-L2-L1, respectively; Philipp et al., 2007; Philipp and Koch, 2009). Importantly, the only difference between the two conditions is whether or not L1 is repeated; in both cases, the language switches from L2 to L1. This “n-2 repetition cost” appears to occur because speakers had to previously suppress L1 to switch into L2, resulting in greater difficulty reactivating the target L1 language.

It is important to recognize that higher proficiency multilinguals and multilinguals with different types of daily language use may experience different cognitive demands (but c.f. Declerck et al., 2015 for evidence that even very proficient multilinguals use inhibition to switch between languages). The influential adaptive control hypothesis proposes that cognitive control demands differ across single-language, dual-language, and code-switching contexts, so multilinguals’ cognitive control mechanisms become tuned for their particular language needs (Green and Abutalebi, 2013). Single-language contexts may require continuous suppression of the unused language, dual-language contexts may require conflict monitoring to detect and correct for instances when the irrelevant language interferes with processing, whereas code-switching contexts may require planning of utterances and flexibility. According to the “weaker links” hypothesis, multilinguals access the associations between lexical representations and semantics/phonology less often than monolinguals (Gollan et al., 2008). These “weaker links” result in greater effort for language processing, especially for languages or representations used infrequently (e.g., a non-dominant L2 in late bilinguals). Critically, although the precise cognitive demands may differ depending on the nature of multilingual language use, multilinguals must engage different cognitive processes than monolinguals because of the need to manage two languages. Regardless of language proficiency or use, it is clear that multilingualism taxes the cognitive system – speakers and listeners must engage effortful cognitive processes to prevent cross-linguistic interference from two or more active languages and/or to retrieve relatively weak lexical representations that are used less frequently.

Recent evidence demonstrates that cognitive control and shifting, both of which have been implicated in multilingual language control, are effortful processes (Shenhav et al., 2017). Participants rate tasks that require more frequent set-shifting as more effortful than those that require less set-shifting (Botvinick et al., 2009). If given a choice, participants prefer tasks that involve less frequent set-shifting and fewer conflict trials (Kool et al., 2010; Schouppe et al., 2014) and give them lower task avoidance ratings (McGuire and Botvinick, 2010). Apparently participants exhibit effort avoidance, the tendency to minimize the amount of work performed (Inzlicht et al., 2018), when faced with cognitive control and shifting tasks. Because these cognitive demands associated with multilingual language control are effortful, multilinguals may need to use cognitive persistence to maintain a consistently high level of effort during routine language use.

The consistent use of cognitive persistence during multilingual language control may enhance the perceived value of successful performance. After working hard to achieve a desired outcome, individuals find the outcome to be more valuable than if the same outcome was achieved with less effort (for review, see Inzlicht et al., 2018). Effort then becomes associated with more rewarding outcomes, which may increase the subjective value of effort itself (Inzlicht et al., 2018). Indeed, neural reward signals are strengthened when effort is exerted or an action is performed to receive the reward, relative to low effort or passive conditions (Elliott et al., 2004; Zink et al., 2004; Hernandez Lallement et al., 2014; Ma et al., 2014). Additionally, effort has been associated with heightened sensitivity to performance outcomes, increasing the stimulus-preceding negativity while participants wait for performance feedback and the amplitudes of the feedback-related negativity and P300 in response to feedback (Wang et al., 2017). This suggests that performance monitoring may be enhanced by the investment of effort. Thus, by continually applying effort during language use, multilinguals may increase the intrinsic value that they place on performing well, thereby reinforcing the need for effort.

### The Neural Bases of Cognitive Persistence

Multilingual language control has routinely been associated with brain activity in the cingulo-opercular network. The cingulo-opercular network consists of the bilateral frontal operculum (inferior frontal gyrus; IFG), anterior insula, and the dorsal anterior cingulate cortex (dACC) / medial superior prefrontal cortex, including supplementary motor (SMA) and pre-supplementary motor areas (pre-SMA) (Dosenbach et al., 2006)^1^. It is classically considered to detect stimulus conflict and implement cognitive control to resolve conflict. This conceptualization of cingulo-opercular function was introduced by Botvinick and colleagues (2001, 1999), who found that the dACC responded to new instances of conflict and seemed to explain adjustments in performance following conflict and errors. Indeed, subsequent research demonstrated that conflict-related dACC/SMA activity predicted increases in lateral prefrontal activity and performance on a subsequent trial (Kerns et al., 2004; Kerns, 2006). The theory that the cingulo-opercular network (and particularly the dACC) is involved in conflict monitoring is highly influential; the original conflict monitoring work (Botvinick et al., 2001) has been cited more than 6500 times on Google Scholar.

Although the conflict monitoring theory is immensely popular, another interpretation of this pattern of results is that dACC activity serves as a signal to try harder in order to improve performance. In other words, the dACC engages cognitive persistence when a task becomes difficult. Teubner-Rhodes et al. (2017) re-conceptualized the dACC as a region that reflects cognitive persistence, the willingness or ability to apply effort to overcome challenges. Similarly, Touroutoglou et al. (2020) proposed that the dACC supports tenacity, which they define as “a persistent pattern of behavior” and a process “by which the costs of effort are devalued, and the value of long-term rewards is emphasized” (p. 13). The idea that dACC enacts cognitive persistence is consistent with functional neuroimaging evidence that errors and response uncertainty engage dACC/pre-SMA and this activity is associated with improving subsequent performance (Carter, 1998; Botvinick et al., 1999; Kerns et al., 2004; Ridderinkhof et al., 2004; Kerns, 2006). Lapses in cingulo-opercular activity precede error commission on sustained attention tasks (Weissman et al., 2006; Eichele et al., 2008), consistent with a downregulation in effort that adversely affects performance. The anterior insula and dACC appear to encode expected effort cost (Botvinick et al., 2009; Prevost et al., 2010), although findings are mixed with regard to whether dACC reflects effort costs alone (Botvinick et al., 2009) or relative to the value of an anticipated reward (Prevost et al., 2010; Shenhav et al., 2013). Even more compelling is the finding that lesions of dACC in rodents and humans result in the avoidance of effortful tasks (Tow and Whitty, 1953; Holec et al., 2014). For instance, rats with dACC lesions were more likely than controls to give up when faced with climbing a barrier to receive a reward (Holec et al., 2014). Importantly, this task involves no informational conflict, but simply requires an increase in effort to surmount an obstacle. Finally, the dACC/pre-SMA and caudate head exhibit greater activity when participants freely select a high-conflict versus a low-conflict Flanker task (Schouppe et al., 2014), implicating these regions in voluntary effort. Together, these findings suggest that the dACC encodes the willingness or the decision to apply effort to overcome difficulty during task execution. Since the relationship between dACC activity and effort has been observed in the absence of stimulus or response conflict, dACC function appears to be a more direct indicator of cognitive persistence than inhibitory control *per se*.

Central to the cognitive persistence interpretation of dACC function is the notion that dACC activity does not merely reflect task difficulty induced by conflict or performance decrements, but also encodes subjective evaluations of the mental cost of applying or sustaining effort and the predicted value of the reward if effort is increased (Teubner-Rhodes et al., 2017; Touroutoglou et al., 2020). Because both the perceived cost of effort and value of the reward are subjective, dACC activity in response to conflict or performance decrements will index individual differences in cognitive persistence. Persistence can be affected by external factors—increasing the value of an external reward should boost dACC activity and consequently persistence. Indeed, providing a higher reward value increases physiological indices of effort and performance on a sustained attention task (Massar et al., 2016). However, when a task does not offer explicit rewards or when reward value is held constant, dACC function will be more closely tied to trait persistence (Holroyd and Umemoto, 2016; Umemoto and Holroyd, 2016). Here, individual differences in effort will be determined in part by intrinsic motivation—how inherently rewarding an individual finds successful performance on a challenging task. Individuals who are motivated to perform well will have higher dACC activity and work harder to succeed than those with lower persistence.

Recent research demonstrates that dACC function may capture individual differences in cognitive persistence. Individuals with growth mindsets who endorse the belief that effort is important for learning show larger performance improvements following errors than those with fixed mindsets (Moser et al., 2011). Importantly, this effect is mediated by a larger dACC-generated error positivity, suggesting that the dACC response to errors underlies effort-related performance boosts. Anhedonia, a symptom of depression characterized by the absence of pleasure, reduces sensitivity to performance feedback in the dACC/pre-SMA (Mies et al., 2013), suggesting that blunted performance monitoring responses in the dACC are associated with decreased motivation. Individuals with higher trait persistence exhibit an increased tendency to choose a high effort versus a low effort task, and greater dACC activity when selecting the higher effort task (Kurniawan et al., 2010). These findings appear inconsistent with evidence that dACC activity when performing a more challenging task was unrelated to the tendency to avoid difficult tasks (McGuire and Botvinick, 2010). However, McGuire and Botvinick (2010) measured global task activity rather than dynamic trial-to-trial activity. It is possible that dACC fluctuations—its sensitivity to errors and response uncertainty and link to subsequent performance—encodes cognitive persistence. Taken together, the evidence suggests that individual differences in persistence and related traits are associated with the extent to which dACC responds to performance and the choice to expend effort.

The effects of task ability and persistence on performance may be confounded in many executive function tasks, making it difficult to determine if differences in performance are due to executive function ability or effort. Recall that persistence depends both on external task demands as well as the intrinsic valuation of effort. Individuals with lower executive function ability may therefore give up more easily because the task is harder for them and would require a greater investment of effort to yield successful performance. Indeed, demand avoidance is especially high for individuals with poor executive function (Kool et al., 2010). Moreover, individuals with high persistence may have similar performance to those with better executive function but lower persistence. To address these issues, Teubner-Rhodes et al. (2017) developed a novel measure of cognitive persistence based on the Wisconsin Card Sorting Test-64 (WCST-64; Kongs et al., 2000) that disentangled the contributions of set-shifting ability and effort to performance. Cognitive persistence was defined as the extent to which individuals over- or underperformed expectations based on their set-shifting ability. This cognitive persistence metric predicted cingulo-opercular function in older adults during a speech recognition-in-noise task. Specifically, cognitive persistence was associated with sensitivity to errors in the dACC/pre-SMA and left IFG as well as the link between dACC/pre-SMA activity and subsequent performance improvements (Teubner-Rhodes et al., 2017); in contrast, set-shifting ability was not associated with cingulo-opercular effects. Thus, when isolated from task ability, persistence is related to the neural instantiation of effort during language control in older adults.

### The Neural Bases of Multilingualism

#### Multilingual Language Control Is Supported by the Cingulo-Opercular Network

Given the evidence that the cingulo-opercular network, in particular the dACC, enacts cognitive persistence, investigating the role of cingulo-opercular regions during multilingual language control is essential to a cognitive persistence account of the multilingual brain during healthy aging. Switching from one language to another activates the bilateral IFG, insula, and dACC/pre-SMA in healthy young adults in production (Garbin et al., 2011; Guo et al., 2011; Abutalebi et al., 2012; De Baene et al., 2015; Branzi et al., 2016; but c.f. Blanco-Elorrieta and Pylkkanen, 2016) and comprehension (Abutalebi et al., 2007; Blanco-Elorrieta and Pylkkanen, 2016). dACC activation during language switching is observed for early, high-proficiency multilinguals (Abutalebi et al., 2007; Garbin et al., 2011; De Baene et al., 2015; Blanco-Elorrieta and Pylkkanen, 2016; Branzi et al., 2016) and sequential multilinguals of varying proficiency levels (Guo et al., 2011; Abutalebi et al., 2012). However, dACC activation is less apparent for switches that occur in natural speech (Blanco-Elorrieta and Pylkkanen, 2017). It is likely that natural speech provides linguistic cues that facilitate switching between languages and lessen demands on the cingulo-opercular system. Outside of the cingulo-opercular network, the dorsolateral prefrontal cortex, inferior and superior parietal lobe, middle temporal gyrus, and head of the caudate have also been implicated in switching languages (Hernandez et al., 2000; Abutalebi et al., 2007; Hernandez, 2009; Garbin et al., 2011; Guo et al., 2011; Branzi et al., 2016).

Language switching and non-linguistic conflict co-activate voxels in the dACC and left IFG (Abutalebi et al., 2012; De Baene et al., 2015). One interpretation of these results is that cingulo-opercular regions are responsible for applying effort across task domains, and they interface with language-switching regions to enact language control. Indeed, Tabassi Mofrad and Schiller (2019) observed that functional connectivity between right inferior parietal cortex and dACC increased when sequential Dutch-English bilinguals switched from L1 to L2. Thus, the cingulo-opercular network may engage and increase crosstalk with language-switching areas in the service of effortful language switching.

Some evidence suggests that the cingulo-opercular network is selectively engaged when switching into a non-dominant language. Speaking and listening in a non-dominant language is thought to be more demanding due to heightened competition from the dominant language (Abutalebi and Green, 2008) and weaker lexical representations associated with less frequent language use (Gollan et al., 2008). In reverse-dominance bilinguals who use their L2 significantly more than their L1, switches into the non-dominant L1 during listening recruited the dACC and the left caudate (Abutalebi et al., 2007). During blocked picture naming, item-specific switches from dominant L1 to non-dominant L2 selectively activated the dACC/pre-SMA in early, sequential German-Italian bilinguals (Branzi et al., 2016). Relatedly, during mixed-language picture naming paradigms, the bilateral dACC/SMA, IFG, and caudate showed elevated activity for L2 compared to L1 in late bilinguals (Abutalebi et al., 2008; Reverberi et al., 2015). Later age of acquisition in Spanish-English bilinguals is associated with greater left IFG activity when naming pictures in L2 (Vaughn et al., 2016). Together, this evidence suggests that the cingulo-opercular network may be involved in boosting effort to facilitate retrieval of representations from a less practiced language. However, Garbin et al. (2011) found that early, balanced Spanish-Catalan bilinguals engaged the dACC/pre-SMA when switching into their preferred language during picture naming but not their less preferred language. While the dACC may enact persistence during effortful language control, its role appears to be modulated by age of acquisition and language proficiency.

The cingulo-opercular network is also involved in other types of effortful language control. Compared to reading, language translation engages the dACC/SMA, left anterior insula, and bilateral striatum in high-proficiency German-English bilinguals (Price et al., 1999). Single-language lexical decision tasks show that interlingual homographs, words that look alike but have different meanings in two languages, activate bilateral IFG and dACC/pre-SMA for late, high-proficiency Dutch-English bilinguals (van Heuven et al., 2008) and late Chinese-Japanese L2 learners (Hsieh et al., 2017). Processing interlingual homographs is challenging because the word form activates conflicting entries across language systems (van Heuven et al., 2008). L2 lexical retrieval during the early stages of word learning in a second language is associated with activity in the left IFG and insula, middle frontal gyrus, and dACC (Raboyeau et al., 2010). Taken together, this evidence suggests that the cingulo-opercular network is involved in multilingual language processing when cross-linguistic conflict or infrequent lexical representations make lexical retrieval effortful.

#### Multilingual Experience Affects the Cingulo-Opercular Network

Multilingualism appears to alter the structure and function of cingulo-opercular regions, demonstrating experience-based plasticity that could potentially protect the brain from detrimental aging effects. The nature of the effect of multilingualism on cingulo-opercular regions likely depends on the age of L2 acquisition. In young children, the dACC is coupled with the fronto-parietal network and only becomes integrated with the cingulo-opercular network over the course of development (D’Souza and D’Souza, 2016). Hearing multiple languages from birth places greater demands on the dACC during a formative time, so that early multilingualism can shape the extent to which dACC integrates with the cingulo-opercular network. In contrast, late multilinguals must learn to speak L2 using existing neural architecture. Although the experience of multilingualism can still affect the brain in these individuals, the basic architecture of their cingulo-opercular network is assumed to be more similar to that of monolinguals. In the subsequent sections, I consider how the cingulo-opercular network is shaped by multilingualism for early and late L2 learners.

Early exposure to multiple languages is associated with increased cortical thickness in the left IFG (in young children; Thieba et al., 2019; but c.f. Klein et al., 2014) and the right ACC (in young adults; Felton et al., 2017). Cingulo-opercular lateralization appears to be altered in early multilinguals. ACC cortical thickness exhibited a rightward asymmetry in Spanish-English bilinguals but had a leftward asymmetry in monolinguals (Felton et al., 2017). When performing task switching, early Spanish-Catalan bilinguals recruited left IFG and insula to a greater extent than monolinguals, whereas monolinguals had higher right IFG and insula activity (Garbin et al., 2010). Early multilingualism also affects the functional consequences of ACC sulcal symmetry, assessed by the parallel presence or absence of the paracingulate sulcus across hemispheres: the Flanker conflict effect was smaller in bilinguals if their ACC sulci were symmetrical as opposed to asymmetrical, while the reverse was true for monolinguals (Cachia et al., 2017).

Early multilinguals may retain increased connectivity with the fronto-parietal network over the course of development. Functional connection strengths from dACC to dlPFC and the striatum increased in early bilinguals and decreased in monolinguals when mentally applying new task rules (Becker et al., 2016). In contrast, resting-state connectivity between the right IFG and dACC was weaker in young multilingual compared to monolingual children (Thieba et al., 2019). This suggests that early multilingualism may alter the developmental trajectory of the dACC, such that the degree to which the dACC decouples from the fronto-parietal network and integrates with the cingulo-opercular network is reduced in multilinguals. Retaining dACC connectivity with the dlPFC may help multilinguals set and maintain task goals, such as mapping a target language to a conversational partner, in dual-language environments.

Early multilingualism may also improve the efficiency of the dACC during cognitive control and language control tasks. Early Spanish-Catalan bilinguals had a smaller switch cost than monolinguals during a task-switching paradigm, but only monolinguals exhibited elevated dACC activity in response to switches (Garbin et al., 2010). Although monolinguals and early Catalan-Spanish bilinguals had comparable reaction times and accuracy during a stop-signal task, monolinguals exhibited greater activity in dACC/pre-SMA than bilinguals when withholding a response (Rodríguez-Pujadas et al., 2014). Following training in a new language, monolinguals were slower and showed greater activation in left ACC and caudate and right dlPFC and SMA when naming newly-learned words than early Spanish-English bilinguals (Bradley et al., 2013). Thus, early multilinguals demonstrate reduced dACC activation while exhibiting performance on cognitive control and language control tasks that is as good as or better than monolinguals. Apparently, early multilinguals can achieve successful performance without engaging the dACC to the same extent as monolinguals.

The effect of multilingualism on cingulo-opercular function depends on age of acquisition, proficiency, and type of language experience. Increased L2 proficiency and experience appears to reduce left dACC volume, activity, and connectivity, while increasing cognitive control. Among late Chinese-English bilinguals, higher L2 proficiency was associated with lower resting-state functional connectivity of left dACC with the left superior and inferior parietal lobe, left IFG, bilateral MFG, and right precuneus (Sun et al., 2019). This lower dACC resting-state connectivity was associated with smaller mixing costs during a Simon-like task, suggesting that greater L2 experience and proficiency improved cognitive flexibility through shifts in left dACC connectivity. Similarly, low-proficiency bilinguals showed elevated activity in the dACC compared to high-proficiency bilinguals during a nonsense-word phonological working memory task, whereas high-proficiency bilinguals exhibited higher activity in the left insula/IFG (Chee et al., 2004). Hours of training as a simultaneous interpreter predicts a reduction in gray matter volume in the bilateral caudate and IFG and left dACC among late multilinguals (Elmer et al., 2014), suggesting the demanding experience of interpreting results in increased pruning within these structures. These results are consistent with findings that L2 usage drives hemispheric specialization of the dACC: Spanish-English bilinguals with lower L1 proficiency (i.e., reverse-dominance bilinguals with greater L2 use) had a larger rightward cortical-thickness asymmetry in the ACC (Felton et al., 2017). Thus, selective pruning of the left dACC and/or increased volume of the right dACC are possible mechanisms for higher dACC efficiency in multilinguals. However, as much of this evidence is from sequential or late multilinguals, it is unclear if brain differences result from L2 usage or if brain differences enable multilinguals to become more proficient in L2.

Taken together, the evidence suggests that early multilingualism and high L2 proficiency reduce involvement of dACC (especially the left dACC) in cognitive control and language control, as well as its baseline connectivity with the cingulo-opercular network. However, the process of learning a second language as an adult appears to have exactly the opposite effect, as increased proficiency is associated with greater dACC engagement and cingulo-opercular connectivity in L2 learners. Pre-training resting-state connectivity between the left anterior insula/IFG and the dACC was higher in healthy young adults who showed larger increases in fluency during spontaneous speech following intensive L2 training (Chai et al., 2016). This suggests that baseline connectivity within the cingulo-opercular network indexes readiness to acquire a second language, perhaps due to increased persistence during language learning. After a year of Spanish language instruction in late L2 learners, the interlingual homograph effect becomes smaller in left IFG and insula and bilateral MFG, but becomes larger in dACC (Grant et al., 2015). This increased engagement of dACC with increased L2 proficiency may reflect preparation of the neural system to apply effort to reduce the experience of interlingual conflict. dACC recruitment may also become more targeted following language learning, responding selectively to homographs, as overall dACC activity during L2 processing decreased over time (Grant et al., 2015). Finally, functional connectivity while making lexical decisions increased between dACC and IFG and dACC and MTG following L2 instruction (Grant et al., 2015). This may indicate that learning a second language as an adult increases the coupling between dACC and regions enacting cognitive control over lexical retrieval.

The evidence regarding the effect of multilingualism on cingulo-opercular function provides an apparent paradox—increased proficiency during L2 learning boosts dACC activity and cingulo-opercular connectivity when language control is required, but high proficiency multilinguals have lower left dACC activity and cingulo-opercular connectivity than those with lower proficiency. This paradox can be explained by the dynamic interplay between persistence and cognitive control: while persistence may drive individuals to engage cognitive control, better cognitive control abilities reduce the need for persistence. As argued above, persistence is enacted by engaging the dACC to apply effort when a task becomes difficult or performance declines. Tight coupling between the dACC and IFG would enable the effective application of cognitive control following dACC-mediated signals for increased effort. Individuals with higher baseline cingulo-opercular connectivity may have higher persistence when learning a second language as an adult, enabling them to achieve higher L2 proficiency. Moreover, as they learn that effort facilitates L2 production and comprehension, they become more likely to engage dACC when they experience linguistic conflict and the coupling between dACC and IFG increases. However, the continued use of frontal control regions to resolve linguistic conflict may improve cognitive control ability. As cognitive control ability improves, the task of managing two languages becomes easier, reducing the need for dACC-mediated persistence. Similarly, early multilinguals engage the dACC to a lesser extent during language and cognitive control because it is less effortful—differential development of conflict resolution mechanisms (e.g., increased reliance on left versus right IFG) enables more efficient control. However, contexts that boost cross-linguistic competition (e.g., dual-language contexts) would still require persistence, even in highly proficient or early multilinguals. The extent to which multilingualism improves persistence or cognitive control thus depends on age of acquisition, proficiency, and type of language use.

Multilinguals with higher persistence are expected to use control more proactively. Using proactive rather than reactive control is typically a more effortful strategy, because it requires keeping cognitive control resources active continuously (Braver, 2012). Mounting evidence suggests that some types of multilingualism may promote proactive cognitive control strategies, perhaps as a result of changes in cingulo-opercular function. Bilinguals exhibit smaller sequential congruency effects on Stroop and Flanker tasks than monolinguals (Grundy et al., 2017; Teubner-Rhodes et al., 2019), indicating that they are proactively applying a consistent level of control across trials. Indeed, early Spanish-English bilinguals were more accurate than monolinguals following congruent Stroop trials (Teubner-Rhodes et al., 2019), suggesting that they maintained effort even when the experience of conflict was low. Source localization of EEG during a Stroop task revealed a significant conflict effect in the dACC for late high-proficiency French-German bilinguals but not French monolinguals (Heidlmayr et al., 2015). Monolinguals demonstrated more bilateral frontal activation and an increased N400 amplitude at the midline for incongruent relative to congruent trials, suggesting that higher dACC activity may help bilinguals proactively avoid downstream conflict effects. Early and late multilingual populations from a variety of language backgrounds and proficiency levels have been found to proactively suppress responding following a non-target cue on the AX-CPT (Gullifer et al., 2018; Liu et al., 2019), suggesting that multilinguals tend to use a proactive control strategy (although early bilinguals also outperform monolinguals on trials requiring reactive control; Morales et al., 2013, 2015). Later L2 acquisition and greater exposure to dual-language contexts were associated with a larger benefit from proactive cues among French-English bilinguals, a pattern that was predicted by stronger ACC-left putamen connectivity (Gullifer et al., 2018). This proactive shift may reflect the increased effort required for language use in individuals with later L2 acquisition and higher exposure to language diversity. Language control is more effortful when learning L2 later in life because L1 representations will be stronger, so processing of L2 will require greater suppression of L1. Similarly, language control is more effortful in dual-language contexts because both languages must be kept active, resulting in greater cross-language interference. Thus, the consistent effort required for language control in these multilingual populations may increase the perceived value of effort itself, resulting in a shift to a more effortful proactive control strategy.

Under the persistence framework, the dACC becomes active when the individual is preparing to enact effort—it turns on when task difficulty is expected or detected and signals to boost effort. Thus, dACC may be engaged proactively or reactively, depending on when effort is prepared. This explains apparent discrepancies in the literature: early, high-proficiency Spanish-English bilinguals demonstrated proactive dACC activity in response to an informative cue about the upcoming target language in a language-switching version of the Rapid Instructed Learning Paradigm (Seo et al., 2018); however, when uninformative cues were introduced to the task, the dACC was engaged reactively during response execution, especially when the target language could not be prepared in advance (Seo and Prat, 2019).

It is clear from the above review that multilingual language experience affects the cingulo-opercular network, which has been implicated in cognitive persistence during challenging tasks. However, the nature of the effect of multilingualism on the cingulo-opercular network depends heavily on the characteristics of the multilingual language experience. Early, high proficiency multilinguals appear to have a more efficient (i.e., less active) dACC, perhaps because improved cognitive control resources make conflict resolution less effortful. In contrast, late L2 learners appear to recruit the dACC more heavily, perhaps because increased effort helps to offset performance decrements when stimulus conflict occurs. Increased persistence, reflected in greater dACC activity in response to conflict, may also enable late L2 learners to *become* bilingual as adults. In the following section, I examine how these effects influence cognitive processing in healthy aging and neurodegenerative disorders.

### Cognitive Aging in Multilinguals

#### Effects of Multilingualism on the Cingulo-Opercular Network in Aging

Older adult multilinguals appear to outperform monolinguals on executive function tasks tapping inhibitory control and shifting abilities. Several studies have found that older adult bilinguals exhibit smaller Simon effects than monolinguals (Bialystok et al., 2004, 2008; Cox et al., 2016). The magnitude of this effect of multilingualism on resolving response conflict increases with age (Bialystok et al., 2004) and remains significant after controlling for childhood IQ and socioeconomic status (Cox et al., 2016). A multilingual advantage among older adults has also been reported on ANT/Flanker, Stroop, manual antisaccade, and task-switching tasks. Late, older adult bilinguals were faster overall and had less skewed RT distributions on incongruent trials on the Flanker task compared to older adult monolinguals (Abutalebi et al., 2015). Age-related performance declines on ANT and Flanker tasks were larger in monolinguals than in bilinguals (Borsa et al., 2018; Del Maschio et al., 2019), indicating that only monolinguals became more susceptible to interference from distractors with age. Across the lifespan, bilinguals produce fewer total errors on the Stroop task (Massa et al., 2020) and have a smaller Stroop effect than monolinguals (Bialystok et al., 2008; Incera and McLennan, 2018), even after accounting for bilinguals’ reduced automaticity of word processing (Bialystok et al., 2008). Bilinguals had superior response suppression and task switching compared to monolinguals in a manual version of the antisaccade task, and this advantage increased with age (Bialystok et al., 2006). Early, high proficiency older adult bilinguals also demonstrated reduced color-shape task switching costs compared to their monolingual peers, while younger bilinguals performed comparably to monolinguals (Gold et al., 2013b). Thus, there is evidence that older adult multilinguals have better executive functioning than monolinguals across a range of tasks and that multilingualism offers some protection from age-related declines in cognitive control.

However, not all findings support a multilingual advantage in cognitive control among older adults. On the classic version of the Stroop task, where participants complete blocks of word reading, congruent color naming, and incongruent color naming, there were no differences in accuracy or reaction time between older adult bilinguals and monolinguals (Kousaie and Phillips, 2012). Borsa et al. (2018) found that healthy middle-aged to older adult monolinguals and early bilinguals performed equivalently on the ANT task. Among sequential, older adult Telugu-English bilinguals, L2 proficiency did not affect performance on ANT, DCCS, Stop-Signal, or numerical Stroop tasks (Mishra et al., 2019), although higher levels of exposure to L2 were associated with larger cuing effects on the ANT, smaller DCCS switch costs, and a smaller Stroop effect. These mixed findings suggest that the effect of multilingualism on cognitive control in healthy aging may depend on the type of language experience or the executive function components being tested—I will return to this idea in section “Sources of Variability in Multilingual Cognitive Aging.”

Structural changes in the cingulo-opercular network may help protect multilinguals from age-related declines in cognitive control. Evidence suggests that volumetric reductions in cingulo-opercular regions are larger in monolinguals than multilinguals during healthy aging. Specifically, Abutalebi et al. (2015) found that late, older adult bilinguals had higher gray matter volume in the bilateral ACC than monolinguals. Moreover, aging was associated with decreased gray matter volume of IFG bilaterally in monolinguals, but only in the left-hemisphere for bilinguals (Borsa et al., 2018). These structural changes in older multilingual adults are accompanied by diminished cognitive decline (e.g., less age-related slowing on the Flanker task; Abutalebi et al., 2015; Del Maschio et al., 2019) and altered structure-function relationships between cingulo-opercular regions and cognitive control (Borsa et al., 2018; Del Maschio et al., 2019). For instance, higher ACC volume was associated with smaller conflict effects on the ANT in middle-aged to older bilinguals but not in monolinguals (Borsa et al., 2018), indicating that ACC is related to cognitive control function during healthy aging for bilinguals only. Additionally, symmetrical ACC sulci were associated with better interference resolution across the lifespan in bilinguals but worse resolution in monolinguals (Del Maschio et al., 2019). Thus, older multilinguals exhibit less age-related decline in cingulo-opercular structures and differential associations between cingulo-opercular regions and cognitive control than monolinguals. These neural changes could underlie multilinguals’ relatively preserved performance on cognitive control tasks during healthy aging.

Cingulo-opercular functional connectivity and efficiency may also protect multilinguals from age-related cognitive declines. Compared to their monolingual peers, older adult bilinguals had less activity in the dACC and left dlPFC and IFG when switching tasks (Gold et al., 2013b). Since the bilinguals also demonstrated smaller switch costs than monolinguals, bilinguals’ reduced recruitment of these regions seems to indicate greater neural efficiency. Older adult bilinguals also exhibited earlier peak latency and/or smaller amplitudes than monolinguals for the dACC-generated N2 waveform when performing Stroop, Simon, and Flanker tasks (Kousaie and Phillips, 2017), suggesting that bilinguals detected conflict earlier and more efficiently. Grady et al. (2015) found that older adult bilinguals had stronger resting-state functional connectivity of the IFG/anterior insula with regions of the frontoparietal network and the right ACC than monolinguals. This is consistent with the idea that cingulo-opercular regions may be more integrated with the frontoparietal network in multilinguals as well as evidence for the rightward lateralization of the ACC in multilinguals.

Multilingualism may delay declines associated with neurodegenerative disorders. Bilinguals are typically diagnosed 3–6 years later than monolinguals with disorders such as Alzheimer’s disease (AD; Bialystok et al., 2007; Craik et al., 2010) and the behavioral variant of fronto-temporal dementia (Alladi et al., 2017). Speaking more than 2 languages may further lower the risk of developing cognitive impairment (Perquin et al., 2013). However, the reported effect of multilingualism on dementia onset is small or non-existent in other studies (Sanders et al., 2012; Clare et al., 2016), leading researchers to conclude that variables such as the number of languages spoken, immigrant status, age of acquisition, and language proficiency may interact with any protective effect of multilingualism (Freedman et al., 2014).

One way that multilingualism may postpone cognitive decline is by offsetting the detrimental effects of brain atrophy on cognitive performance. Indeed, older adult bilinguals seem to maintain comparable cognitive function as monolinguals in the face of greater declines in fiber tracts that support frontal connectivity (Gold et al., 2013a) and AD-associated deterioration in the medial temporal lobe (Schweizer et al., 2012). This so-called “cognitive reserve” may be supported by changes in cingulo-opercular structure and function. In bilinguals with Alzheimer’s Disease, higher cortical thickness in the left dACC was associated with more equal language proficiency and with more accurate picture naming in the non-dominant language (Smirnov et al., 2019). Middle-aged to older adult bilinguals with Huntington’s Disease who had more equal use of their languages had increased glucose metabolism in the dACC and left anterior insula than those with less equal language use (Martinez-Horta et al., 2019). More equal language use was also associated with less Stroop interference and faster performance on the TMT-B, suggesting that higher dACC glucose metabolism may have staved-off declines in inhibitory control and set-shifting due to Huntington’s Disease. Taken together, the evidence suggests that balanced usage of more than one language alters cingulo-opercular functionality in healthy aging and dementia while reducing or postponing cognitive decline. However, findings of the effect of multilingualism on cognitive aging have been inconsistent. The next section explores how task impurity and individual differences in sociolinguistic factors have contributed to mixed evidence in the literature.

### Sources of Variability in Multilingual Cognitive Aging

There are two major sources of variability that could impact effects of multilingualism on cognitive control during aging: 1) variability in bilingual language experience and 2) differences in the tasks used to measure executive function. Baum and Titone (2014) highlight the heterogeneity of the concept “bilingualism,” which may include individuals who vary considerably in age of acquisition, L2 proficiency, and frequency/type of language use. They argue that researchers need to examine how language experience (rather than “bilingualism” *per se*) affects neural systems to understand how neuroplasticity may give rise to improved cognitive control processes in aging multilinguals. The executive function tasks typically used in multilingual aging research often have poor convergent validity (Paap and Sawi, 2014), potentially limiting their utility in assessing individual differences in cognitive control. Moreover, different tasks may tap into distinct processes, such as inhibitory control, updating, and shifting (Miyake et al., 2000; Friedman and Miyake, 2017), which could be differentially affected by multilingualism. This issue is exacerbated by the confound between cognitive persistence and task ability on many executive function tasks (Teubner-Rhodes et al., 2017). In the following sections, I discuss how task and linguistic variability may influence the effect of multilingualism on cognitive aging and provide recommendations for future research.

#### Task Variability

The effects of multilingualism on cognitive control in older adults are often inconsistent across different tasks. Massa et al. (2020) found that older, late French-Italian bilinguals had more interference from letter-number switching on the TMT-B than monolinguals, while they were more accurate on Stroop. Thus, these bilinguals demonstrated better verbal inhibitory control but *worse* set shifting than monolinguals. Older adult English-French bilinguals were generally more accurate than monolinguals on the Flanker task, they were faster and more accurate than monolinguals on incongruent but not congruent Stroop trials, and they performed no differently than monolinguals on the Simon task (Kousaie and Phillips, 2017). Performance on these tasks was not correlated for either bilinguals or monolinguals. Similarly, German-English multilingual children had smaller Flanker but not Simon effects than L2 learners, and their conflict effects were uncorrelated (Poarch, 2018; Poarch and van Hell, 2019). On the surface, these results seem to suggest that these tasks tap different components of executive function. However, since Flanker, Stroop, and Simon are all tasks that putatively assess inhibitory control, these results raise concerns about the convergent validity and reliability of the tasks typically used to assess executive functions (see Poarch and Krott, 2019 for a comprehensive discussion of the lack of standardization of these tasks across studies).

One source of variability across tasks is the type of conflict that their stimuli elicit. For instance, the Flanker task involves distractor interference from irrelevant flanking arrows (Friedman and Miyake, 2004). Selective attention to the center arrow may help participants ignore the irrelevant distractors. In contrast, the Stroop task involves conflict between multiple, activated linguistic representations (e.g., the word “blue” and the ink color “red”) that compete for selection. This representational conflict is separable from and may involve different conflict resolution mechanisms than the response conflict between incompatible button-presses associated with each color (Milham et al., 2001; van Veen and Carter, 2005). Although these tasks may involve a common inhibitory control function (Friedman and Miyake, 2004), each executive function task involves stimulus-specific perceptual and processing demands (Miyake et al., 2000; Friedman and Miyake, 2004). Thus, the contribution of these separable subprocesses to performance may mask any shared variance attributable to inhibitory control ability.

This issue of task impurity is exacerbated by the difficulty of separating the effects of effort and cognitive control on performance. As described in sections “What Is Cognitive Persistence?” and “The Neural Bases of Cognitive Persistence,” individual differences in trait persistence will improve performance on challenging tasks, including those that involve set shifting, inhibitory control, and language control functions. Moreover, because persistence is more beneficial under difficult task conditions, its effect on performance may appear quite similar to that of executive functions; namely, high persistence will result in better performance on difficult conflict or switching trials while having less of an effect on easier, non-conflict trials. In this manner, multilinguals and monolinguals with high persistence but low-to-medium cognitive control may exhibit performance that looks like individuals with higher cognitive control. Moreover, the relationship between persistence and the execution of effort during task performance is not straightforward. Although individual differences in trait persistence may be relatively stable, the amount of effort that an individual applies to a task also depends on how difficult they find the task to be. This difficulty-level is determined by the individual’s ability to perform each of the perceptual and cognitive processes that the task requires. Finally, the contribution of persistence to performance may become greater with age, as tasks become more challenging due to declining perceptual abilities (e.g., hearing loss and visual impairments), processing speed, and cognitive control functions.

**FIGURE 2:**
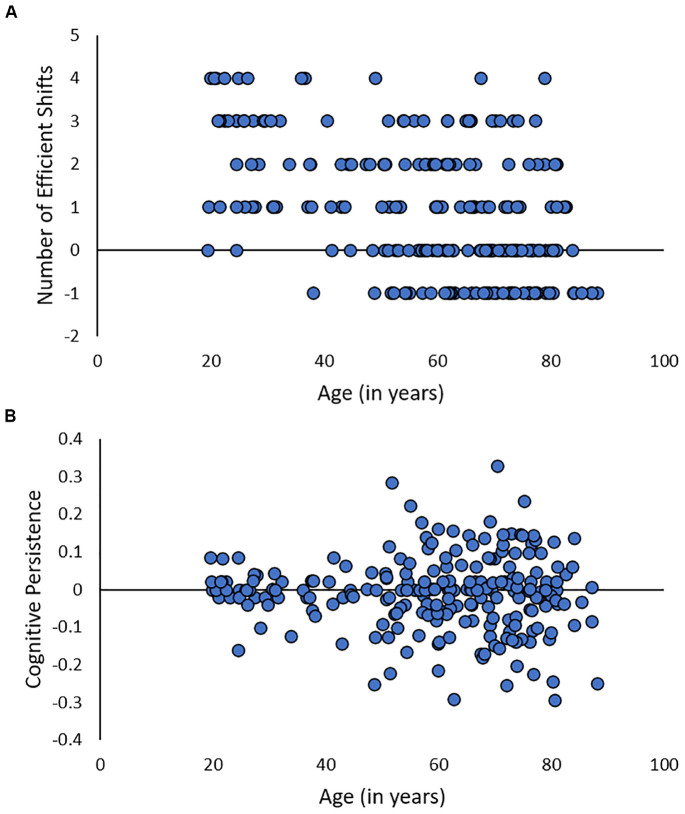
Unpublished data from Teubner-Rhodes et al. (2017) showing the relationship between age and number of efficient shifts **(A)** or cognitive persistence **(B)** on the WCST-64.

Given the inherent confound between persistence and cognitive control, how can researchers systematically investigate the effects of multilingualism on persistence and cognitive control ability in aging? It is essential to control for individual differences in cognitive persistence when assessing performance on cognitive control tasks. Only by separating performance into persistence and cognitive control function can researchers evaluate the effects of multilingualism on each of these components. One approach is to use the method developed by Teubner-Rhodes et al. (2017) to measure both persistence and set-shifting ability from the widely-used WCST-64 task (Kongs et al., 2000). Advantages to this approach are that researchers can use a single task to compare persistence and set-shifting in multilinguals and monolinguals and that it removes the confound between persistence and set-shifting, as the metrics are orthogonal by design. Because persistence on the WCST-64 is related to cingulo-opercular function in older adults (Teubner-Rhodes et al., 2017), it is a plausible candidate for protecting the multilingual brain from the detrimental effects of aging. Interestingly, unpublished data from the cross-sectional sample of 230 adults across the lifespan in Teubner-Rhodes et al. (2017) shows that while number of efficient shifts on the WCST-64 is significantly negatively correlated with age (*Kendall’s τ* = −0.34, *p* < 0.001), persistence is unrelated to age (*Kendall’s τ* = −0.02, *p* < 0.58; Figure 2). Because persistence does not appear to be susceptible to cognitive aging effects, individuals with higher persistence may be able to offset performance decrements due to age-related declines in set-shifting ability. However, research-to-date has not investigated the effect of multilingualism on persistence versus set-shifting on the WCST-64.

There are several other established behavioral persistence tasks that researchers can use to control for effort when examining individual differences in performance on executive function tasks. These tasks typically assess persistence by measuring how long participants choose to work on a very difficult or impossible task before giving up. Examples include the computerized Mirror-Tracing Persistence Task (MTPT; Quinn et al., 1996; Daughters et al., 2005a), in which participants attempt to trace the outline of a complex shape that is mirror-reversed without making any errors; the persistence-version of the Paced Auditory Serial Addition Test (PASAT; Lejuez et al., 2003), which presents numbers in rapid succession and requires participants to add the current number to the preceding one; and the Anagram Persistence Task (APT; Eisenberger and Leonard, 1980; Quinn et al., 1996), which asks participants to re-arrange sets of scrambled letters to make words, some of which are unsolvable. Performance on behavioral persistence tasks predicts adherence to addiction disorder treatment programs (Daughters et al., 2005a,b,c), demonstrating their relevance for evaluating maintenance of effort in the face of real-world stressors. Consistent with the premise that persistence affects performance on cognitive tasks, time spent on unsolvable anagrams during the APT is modestly, positively correlated with a factor measuring general intelligence (Gignac and Wong, 2018). Taken together, these findings suggest that the amount of time participants spend trying to complete a cognitively-demanding task is a valid assessment of their willingness to persist to overcome a mental challenge.

I recommend that researchers incorporate one or more of these persistence metrics (WCST-64, MTPT, PASAT, APT) into studies examining the effects of multilingualism during aging. Not only will these tasks allow researchers to directly investigate the effects of multilingualism on persistence, but they will also enable them to estimate variability in performance on executive function tasks due to persistence versus the executive function of interest. In this way, researchers can adopt a more targeted approach to study how different types of multilingualism affect component processes that are hypothesized to be enhanced by specific linguistic experiences.

#### Linguistic Variability

The evidence reviewed in section “Cognitive Persistence in Multilingualism” demonstrates that the experience of multilingualism affects the cingulo-opercular network, which I have argued is responsible for cognitive persistence, the willingness or ability to implement mental effort. Compared to monolinguals, multilinguals exhibit structural and functional differences in cingulo-opercular regions across the lifespan (Gold et al., 2013b; Abutalebi et al., 2015; Grady et al., 2015; Kousaie and Phillips, 2017; Borsa et al., 2018; Del Maschio et al., 2019). In older adults, these differences have been associated with superior performance on challenging tasks requiring cognitive control (Gold et al., 2013b; Abutalebi et al., 2015; Kousaie and Phillips, 2017) and may delay the onset of cognitive symptoms in some types of dementia (Bialystok et al., 2007; Craik et al., 2010; Perquin et al., 2013; Alladi et al., 2017). However, the nature and extent of multilingualism-induced changes in the cingulo-opercular system appears to depend on socio-linguistic factors (Green and Abutalebi, 2013; Baum and Titone, 2014; Freedman et al., 2014), including age of L2 acquisition, language proficiency, number of languages spoken, and possibly education level. This is critically important because the nature of changes in the cingulo-opercular system will determine the extent to which multilingualism affects persistence versus cognitive control.

To the extent that multilingualism improves cognitive control, then it lessens the effort that is required to perform cognitive control tasks, including management of competing language systems during language use. If effort is not substantially taxed during language use, then persistence is not expected to improve. However, multilinguals with relatively lower cognitive control will need to use effort to enact language control, and so are expected to become more persistent over time. Additionally, multilinguals who are in more cognitively demanding linguistic environments (e.g., dual-language contexts) may also experience improvements in persistence.

Simultaneous and early sequential multilinguals appear to demonstrate greater connectivity between the dACC and the fronto-parietal network (Becker et al., 2016) and reduced coupling between the dACC and right IFG (Thieba et al., 2019). These changes are accompanied by increased left IFG activity and decreased dACC activity in response to conflict (Garbin et al., 2010; Rodríguez-Pujadas et al., 2014). I propose that these neural changes enable multilinguals to enact cognitive control more successfully than monolinguals in the face of neural declines associated with healthy aging and the early stages of dementia. Prior work has shown that older adults over-recruit frontal regions on executive function and working memory tasks compared to younger adults (Schneider-Garces et al., 2010; Vermeij et al., 2012; for review see Reuter-Lorenz and Cappell, 2008), especially for relatively low task demand levels (but cf. Jamadar, 2020). This effectively limits older adults’ dynamic range of neural activity (Schneider-Garces et al., 2010; Kennedy et al., 2015), such that frontal activity hits ceiling as task demands increase. According to the “compensation-related utilization of neural circuits hypothesis” (Reuter-Lorenz and Cappell, 2008), this increased frontal engagement enables older adults to achieve similar levels of performance as younger adults in low demand conditions, but their performance declines in higher demand conditions as their frontal activation maxes out. Thus, early multilinguals’ reduced dACC activity in response to conflict may preserve the dynamic functional range of the dACC during healthy aging, enabling them to outperform older monolingual adults in high demand conditions.

The picture is even more complicated for late multilinguals: the early developmental trajectory of the cingulo-opercular network in late multilinguals cannot be affected by multilingual language experience, because individuals have not yet learned a second language. Pre-existing neural differences may in part determine which individuals successfully learn a second language, but the experience of acquiring a second language later in life could still lead to changes in the cingulo-opercular network. Although the direction of causality is unclear, the process of acquiring proficiency in late L2 learners is associated with increased connectivity between dACC and left IFG/insula and more targeted dACC activation in response to cross-language conflict (Grant et al., 2015; Chai et al., 2016). As the ability of the dACC to track difficult conditions is important for cognitive persistence (Holroyd and Umemoto, 2016; Teubner-Rhodes et al., 2017; Touroutoglou et al., 2020), late multilinguals may be better able to initiate and sustain effort on demanding tasks than monolinguals, especially as sensory and cognitive functions decline with age.

Table 1 illustrates the hypothesized effects of different types of early and late multilingualism on the organization and responsivity of the cingulo-opercular network. This is not an exhaustive list of multilingual language experiences, but is intended to show how the effects of classic early (L2 acquisition before primary school with balanced or nearly balanced proficiency and usage) and late multilingualism (L2 acquisition as adult with higher proficiency and usage of L1 than L2) are differentially modulated by certain, more demanding multilingual contexts. The classic early multilingual speaker is hypothesized to have a fundamental restructuring of the cingulo-opercular network from an early age, including increased integration of dACC with fronto-parietal regions compared to monolinguals. They are hypothesized to apply cognitive control more efficiently than monolinguals. The extent of cingulo-opercular reorganization and its effect on cognitive control depends on L2 proficiency and usage, with smaller effects in individuals with lower proficiency (either resulting from less balanced usage or reflecting a limiting factor that caused their reduced proficiency) and larger effects in multilinguals who are frequently in demanding dual-language contexts that require switching languages to communicate with different conversational partners. Late multilinguals would not experience a fundamental reorganization of the cingulo-opercular network but may still exhibit small changes in structure volume and connectivity (e.g., rightward lateralization of dACC; Elmer et al., 2014; Sun et al., 2019; increased dACC-left IFG connectivity; Grant et al., 2015; Chai et al., 2016) due to the cognitive control demands of using L2, albeit to a lesser extent than early multilinguals. As in early multilinguals, the extent of these effects is expected to increase with greater L2 usage and proficiency.

**TABLE 1:** Effects of early and late multilingualism on the cingulo-opercular network, cognitive control, and cognitive persistence.

	Reorganization of cingulo-opercular network and benefit to cognitive control		Heightened dACC responsivity and benefit to cognitive persistence	
Speaker type	Early multilingual	Late multilingual	Early multilingual	Late multilingual
Lower proficiency	Low	None	Moderate	None
Classic speaker	Moderate	Low	None	Moderate
Dual-language context	High	Moderate	Low	High

*Reorganization of the cingulo-opercular network refers to changes in regional volume, lateralization of structure and function, and connectivity that are associated with superior or more efficient cognitive control, whereas heightened dACC responsivity refers to a tighter coupling between activity and task demand/performance that is associated with cognitive persistence.*

The classic early multilingual is hypothesized to have similar levels of cognitive persistence as monolinguals because L2 usage is not effortful under most circumstances. However, more demanding linguistic environments may still tax cognitive persistence even in early multilinguals. L2 processing is more effortful for early multilinguals with lower proficiency due to weaker L2 lexical representations and greater interference from the dominant L1. Early multilinguals in dual-language contexts also need to exert effort to enact language switching. These additional processing demands are expected to heighten ACC responsivity to difficult conditions (e.g., infrequent L2 representations, interlingual homophones, language switches) and improve cognitive persistence relative to the classic early multilingual. However, these changes will be larger in early multilinguals with lower proficiency because the hypothesized cognitive control benefit in multilinguals with frequent dual-language context experience will partially offset the experience of effort.

The hypothesized effect of multilingualism on dACC sensitivity and cognitive persistence follows a different pattern for late multilinguals. Reduced baseline dACC connectivity with left IFG/insula is expected to yield poor cognitive persistence in some L2 learners, limiting gains in language proficiency—these late L2 learners will not experience significant changes in organization or reactivity of the cingulo-opercular network and are functionally equivalent to monolinguals. The effort required to acquire and use L2 as an adult is predicted to enhance dACC sensitivity to performance difficulty and conflict in the classic late multilingual, resulting in superior cognitive persistence. Effects will be even larger in late multilinguals who frequently encounter dual-language contexts, due to the additional demands associated with language switching.

Researchers can test these hypotheses by including one or more measures of cognitive persistence (e.g., MTPT, PASAT, APT, or WCST-64) when testing the effect of multilingualism on executive function. For instance, consider an aging study examining the effect of L2 proficiency on the Flanker conflict effect (incongruent response times – congruent response times) and the MTPT in early multilinguals. Early multilinguals with lower proficiency are hypothesized to have lower cognitive control but higher cognitive persistence than those with higher proficiency (see Table 1). Thus, in younger early multilinguals, lower proficiency is predicted to be associated with a larger conflict effect (poorer cognitive control) and more time spent trying to trace the mirror-reversed figure in the MTPT (higher persistence). Recall that the benefit of cognitive persistence on performance on cognitive control tasks like Flanker is thought to increase with age, as the task becomes more difficult. As a relatively stable trait, cognitive persistence contributes more to performance in older adults because it can compensate for declines in sensory and cognitive processes that make the task more challenging. Thus, multilinguals with higher persistence would exhibit slower age-related increases in the magnitude of the Flanker conflict effect. Including assessments of cognitive persistence in multilingual aging experiments thus has the potential to illuminate separate effects of multilingualism on cognitive persistence and executive function and identify a protective mechanism against cognitive aging.

## Conclusion

Multilingual language control engages the cingulo-opercular network and appears to induce lifelong changes in the structure and function of this system. Cingulo-opercular brain function has repeatedly been linked to cognitive persistence, or the application of effort on mentally challenging tasks, which is an important element of performance on cognitive control tasks. Thus, a promising avenue for future research is to explore the effects of multilingualism on cognitive persistence. Multilingualism may preserve the dynamic range of cingulo-opercular activity and the link between dACC function and performance during healthy aging. Cognitive persistence may enable multilingual older adults to outperform their monolingual peers by offsetting age-related decrements in cognitive control. Because multilingualism interacts with sociolinguistic factors, future research should use tasks or approaches that differentiate cognitive functions (e.g., persistence, inhibitory control, shifting) that are tapped by different types of multilingual language experience.

## Data Availability Statement

The raw data supporting the conclusions of this article will be made available by the authors, without undue reservation.

## Author Contributions

ST-R was responsible for all conceptualization, analyses, and writing for this work.

## Conflict of Interest

The author declares that the research was conducted in the absence of any commercial or financial relationships that could be construed as a potential conflict of interest.

## Footnotes

1 The region typically defined as the dACC in the cingulo-opercular network extends dorsally into the medial superior prefrontal cortex. Authors often use the term dACC or rostral cingulate zone to describe functional results that also include pre-SMA and/or SMA. I will use dACC when referring to this region more broadly, but in the interest of anatomical specificity, I will use pre-SMA and SMA when discussing functional results localized to these areas. Note that these regions are all part of the cingulo-opercular network and are thought to serve similar functions.
